# Evidence-based management of gastrointestinal diseases

**DOI:** 10.1093/gastro/gou092

**Published:** 2015-02-02

**Authors:** J. Thomas Lamont

**Affiliations:** Division of Gastroenterology, Beth Israel Deaconess Medical Center, Harvard Medical School, DA-501, 330 Brookline Avenue, Boston, MA 02215, USA. Tel: +1-617-667-8377; Fax: +1-617-667-2767; Email: jlamont@bidmc.harvard.edu

This special issue of *Gastroenterology Report* is directed at “Evidence-based management of gastrointestinal (GI) diseases”. Gastroenterologists are now using a range of diagnostic tests and therapies that were largely unavailable twenty years ago. Ours is a fast-moving field, with innovations being developed at a rapid pace.

Included in this issue are authoritative reviews on how to manage a wide variety of conditions that affect the GI and biliary tracts using both established and emerging technologies, with emphasis on evidence to support the best practices and to achieve the best possible outcomes for our patients; for example, much has changed as regards our diagnostic accuracy in celiac disease. In the past 20 years the field has been revolutionized by the development of the current diagnostic test of choice: serum IgA antibody against tissue transglutaminase (IgA-tTG). This assay replaced the earlier ‘gold standard', the anti-endomysial antibody (EMA). As reviewed in detail by Castillo *et al.* in this issue [1], IgA-tTG is the screening test of choice for detecting celiac disease in practice, with a specificity and sensitivity above 95%. Importantly, the incidence of celiac disease in Europe and North America approaches 1% of the population, but many patients are undiagnosed because they do not exhibit classic signs or symptoms of disease such as chronic diarrhea, weight loss, malabsorption or anemia.

Bonfrate and colleagues provide a useful and detailed review of non-invasive breath tests using ^13^C, a stable and non-radioactive isotope, to assess liver metabolic function and gastric emptying [2]. These tests can be used safely in children and during pregnancy to provide useful data for clinical research or for patient management; for example, breath excretion of ^13^C phenylalanine can predict post-operative hepatic complications and post-resection liver regeneration. Similarly, sophisticated breath tests can non-invasively and safely measure hepatic mitochondrial and microsomal function.

One of the most difficult clinical situations is the question of whether a biliary structure is benign or malignant. Singh *et al.* provide an authoritative and practical guide comparing the use of conventional techniques [endoscopic retrograde cholangiopancreatography (ERCP), endoscopic ultrasonography (EUS) with fine needle aspiration (FNA)], and emerging approaches (intraductal ultrasound, cholangioscopy and confocal biliary microscopy) to differentiate neoplastic bile duct strictures [3]. These latter techniques still require refinement and further experience, but will probably be added to our diagnostic algorithms in the coming years.

A much-feared complication of ERCP is pancreatitis, which can range from mild pain with hyperamylasemia, to catastrophic pancreatitis with phlegmon or abscess formation. Thaker *et al.* provide an evidence-based approach to the causes and clinical features of this condition, and provide some guidelines for avoiding post-ERCP pancreatitis (PEP) including patient selection, use of guidewire, avoidance of multiple cannulation attempts or injections and the use of a temporary stent in the pancreatic duct in high-risk patients [4]. Rectal suppositories containing non-steroid anti-inflammatory drugs (NSAIDS) after ERCP to prevent PEP has now been adopted in many high-volume ERCP centers.

Refractory gastroesophageal reflux disease (GERD) is increasingly recognized in patients with reflux esophagitis, who continue to have symptoms despite appropriate therapy with proton pump inhibitors (PPIs). Subramanian *et al.* outline a logical and practical plan for optimizing the management of these patients. Increasing the dose of PPI to twice daily or switching to another type of PPI is successful in some patients [5]. Truly refractory patients may benefit from referral to discuss surgical options. Also discussed are newer endoscopic approaches to refractory GERD.

Refractory GERD, and acid reflux in general, are known risk factors for Barretts esophagus (BE), or metaplasia of the squamous epithelium lining the lower end of the esophagus. Yachimski *et al.* provide a scholarly review of the evidence base to support screening and surveillance of BE, with the goal of preventing its progression to adenocarcinoma [6]. In the past decade a shift has occurred in treating high-grade dysplasia or cancer *in situ* in BE. Evidence supports the benefit of endoscopic radiofrequency ablation to treat high-grade dysplasia and mucosal cancer. Despite improvements in the detection and management of BE, the incidence of esophageal adenocarcinoma continues to climb, highlighting the need for better screening and prevention.

One of the fastest-moving areas in gastroenterology is biological treatment of inflammatory bowel disease (IBD), a new form of therapy based on monoclonal antibodies that inhibit inflammation, reviewed here by Moss [7]. The prototype drug in this field was infliximab, a TNF inhibitor with activity against Crohn's disease, ulcerative colitis, psoriasis and rheumatoid arthritis. The use of biologics for IBD requires careful patient evaluation to eliminate the risk of opportunistic infections such as tuberculosis, viral infections including hepatitis and cytomegalovirus (CMV), as well as infusion reactions and allergic reactions. Biological agents are often given with immunosuppressive drugs, including azathioprine or methotrexate, to prevent antibody formation, a common cause of loss of therapeutic effect after initial response. Biological therapy for IBD and other autoimmune diseases is likely to change our therapeutic approach to millions of patients in the next few years.
